# Dual targeting of peroxisomal proteins

**DOI:** 10.3389/fphys.2013.00297

**Published:** 2013-10-18

**Authors:** Julia Ast, Alina C. Stiebler, Johannes Freitag, Michael Bölker

**Affiliations:** ^1^Department of Biology, Philipps University MarburgMarburg, Germany; ^2^LOEWE Centre for Synthetic Microbiology (SYNMIKRO)Marburg, Germany; ^3^LOEWE Excellence Cluster for Integrative Fungal Research (IPF)Marburg, Germany

**Keywords:** peroxisomes, protein import, alternative splicing, ribosomal read-through, glycolysis

## Abstract

Cellular compartmentalization into organelles serves to separate biological processes within the environment of a single cell. While some metabolic reactions are specific to a single organelle, others occur in more than one cellular compartment. Specific targeting of proteins to compartments inside of eukaryotic cells is mediated by defined sequence motifs. To achieve multiple targeting to different compartments cells use a variety of strategies. Here, we focus on mechanisms leading to dual targeting of peroxisomal proteins. In many instances, isoforms of peroxisomal proteins with distinct intracellular localization are encoded by separate genes. But also single genes can give rise to differentially localized proteins. Different isoforms can be generated by use of alternative transcriptional start sites, by differential splicing or ribosomal read-through of stop codons. In all these cases different peptide variants are produced, of which only one carries a peroxisomal targeting signal. Alternatively, peroxisomal proteins contain additional signals that compete for intracellular targeting. Dual localization of proteins residing in both the cytoplasm and in peroxisomes may also result from use of inefficient targeting signals. The recent observation that some *bona fide* cytoplasmic enzymes were also found in peroxisomes indicates that dual targeting of proteins to both the cytoplasm and the peroxisome might be more widespread. Although current knowledge of proteins exhibiting only partial peroxisomal targeting is far from being complete, we speculate that the metabolic capacity of peroxisomes might be larger than previously assumed.

## Introduction

Peroxisomes are near-ubiquitous eukaryotic organelles that have been first described as microbodies in murine kidney-cells (Rhodin, 1954). Later, these organelles were shown to contain enzymes involved in the turnover of hydrogen peroxide (H_2_O_2_), which gave rise to the term peroxisomes (deDuve and Bauduin, 1966). One of the major sources of H_2_O_2_ is β-oxidation of fatty acids that occurs in peroxisomes throughout the eukaryotic kingdoms (Poirier et al., 2006). Beside these common tasks of fatty acid degradation and peroxide detoxification, peroxisomes exhibit a wide variety of other metabolic functions (Nyathi and Baker, 2006; van der Klei et al., 2006; Wanders and Waterham, 2006). A high degree of functional and metabolic specialization in different organisms even led to specific naming of peroxisomal subtypes. In plants and fungi, glyoxysomes harbor enzymes of the glyoxylate cycle, which is required for the anabolic use of acetyl-CoA as carbon source (Breidenbach and Beevers, 1967; Zimmermann and Neupert, 1980; Kionka and Kunau, 1985). In trypanosomes, the majority of glycolytic enzymes reside in specialized peroxisomes called glycosomes (Opperdoes and Borst, 1977). Filamentous ascomycetes contain “Woronin bodies” that play a mechanical role and seal septal pores (Jedd and Chua, 2000). In spite of their obvious functional and metabolic diversity all types of peroxisomes share a highly conserved import system for their matrix proteins (Gabaldon, 2010). Import is mediated by peroxisomal targeting sequences (PTS), that reside either at the C-terminus (PTS1) or at the N-terminus (PTS2) of proteins (Rucktäschel et al., 2011). C-terminal PTS1 motifs consist of about 12 amino acids that contain at the very end a characteristic tripeptide derived from the prototype sequence SKL (Gould et al., 1987, 1989; Brocard and Hartig, 2006). The commonly used consensus motif for C-terminal tripeptides is (S/A/C)-(K/R/H)-(L/M), while some studies suggest a more degenerated consensus (Lametschwandtner et al., 1998; Reumann et al., 2007). A few proteins contain internal motifs acting as PTS (Peterson et al., 1997; Klein et al., 2002; Gunkel et al., 2004; Oshima et al., 2008; Galland et al., 2010). PTS1 containing proteins are recognized by the cytoplasmic receptor Pex5 and are imported into peroxisomes in their fully folded, oligomeric and even cofactor bound form (Brocard et al., 1994; Glover et al., 1994; McNew and Goodman, 1994). A minority of proteins contains an N-terminal PTS2-motif, which is recognized by the soluble receptor Pex7 (Swinkels et al., 1991; Marzioch et al., 1994; Rucktäschel et al., 2011). PTS2-motifs exhibit the consensus sequence (R/K)(L/V/I)-X_5_-(H/Q)(L/A) (Petriv et al., 2004). Interestingly, some species completely lack the PTS2 import pathway (Motley et al., 2000; Gonzalez et al., 2011). Some proteins have been described that lack any detectable PTS-motifs but are nevertheless found in peroxisomes. A quite unusual way to achieve peroxisomal import of proteins without PTS is “piggy-backing”, since proteins can also be imported as oligomers (Glover et al., 1994; McNew and Goodman, 1994; Yang et al., 2001). It has been demonstrated that peroxisomal import of the copper containing superoxide dismutase (SOD) is mediated via interaction with a chaperone that harbors a PTS1 (Islinger et al., 2009).

**Figure 1:**
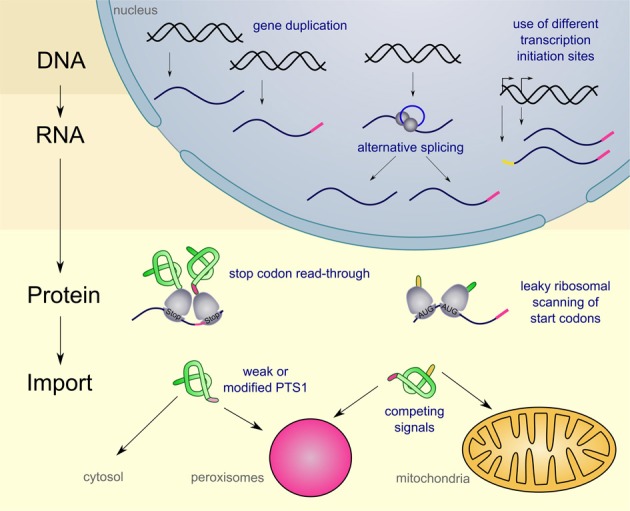
**Schematic overview on the diversity of mechanism leading to dual targeting of peroxisomal proteins**. Peroxisomal targeting signals are indicated in pink, while mitochondrial signal sequences are indicated in yellow. For further explanations see text.

For several peroxisomal proteins dually targeted isoforms have been described. These isoforms execute the same or a similar function in at least one other place (for an overview see Table 1). In general, cells can use various mechanisms to achieve dual or multiple targeting of proteins. One of the best systems studied are mitochondrial proteins some of which occur also in other cellular compartments (for review Yogev and Pines, 2011). Here, we address the diversity of mechanisms to mediate dual targeting of peroxisomal proteins and distinguish five different strategies (see Figure 1): (1) gene duplication, (2) generation of alternative transcripts from single genes, (3) leaky scanning of start and stop codons, (4) competition between multiple targeting signals (5) partial peroxisomal localization of proteins, since the targeting signal is either modified or inefficient.

**Table 1:** **Overview on peroxisomal proteins with dual localization**.

**Enzyme**	**Organism**	**Mechanism for dual localization^a^**	**Localization**	**References**
Malate dehydrogenase	*S. cerevisiae*	Gene duplication	Cyt, Mito, Pex	McAlister-Henn and Thompson, 1987; Minard and McAlister-Henn, 1991; Steffan and McAlister-Henn, 1992
NADPH-dependent isocitrate dehydrogenase	*S. cerevisiae*	Gene duplication	Cyt, Mito, Pex	Haselbeck and McAlister-Henn, 1991; Henke et al., 1998; Loftus et al., 1994; van Roermund et al., 1998
Citrate synthase	*S. cerevisiae*	Gene duplication	Mito, Pex	Kim et al., 1986; Lewin et al., 1990; Rosenkrantz et al., 1986
NADPH-dependent isocitrate dehydrogenase	*A. nidulans*	Alternative transcription initiation sites	Cyt, Mito, Pex	Szewczyk et al., 2001
Malate dehydrogenase	*Y. lipolytica*	Differential splicing	Cyt, Pex	Kabran et al., 2012
6-phosphogluconate dehydrogenase	*C. albicans*	Differential splicing	Cyt, Pex	Strijbis et al., 2012
Hydroxypyruvate reductase	Cucurbita sp. (cv. Kurokawa Amakuri Nankin)	Differential splicing	Cyt, Pex	Hayashi et al., 1996
Glyceraldehyde-3-phosphate dehydrogenase (GAPDH)	*U. maydis*	Differential splicing	Cyt, Pex	Freitag et al., 2012
Phosphoglycerate kinase (PGK)	*A. nidulans*	Differential splicing	Cyt, Pex	Freitag et al., 2012
Phosphoglycerate kinase (PGK)	*U. maydis*	Ribosomal read-through	Cyt, Pex	Freitag et al., 2012
Triose phosphate isomerase (TPI)	*U. maydis*	Ribosomal read-through	Cyt, Pex	Freitag et al., 2012
Iron-containing superoxide dismutase	*L. polyedrum*	Alternative start codons	Plas/Mito, Pex	Bodyl and Mackiewicz, 2007
70-kDa heat shock protein	*Citrullus vulgaris*	Alternative start codons	Pex, Plas	Wimmer et al., 1997
3-Hydroxy-3-methylglutaryl coenzyme A lyase	*H. sapiens* (liver cells)	Multiple targeting signals	Mito, Pex	Ashmarina et al., 1999
Type II NAD(P)H dehydrogenase	*A. thaliana*	Multiple targeting signals	Mito, Pex	Carrie et al., 2008; Xu et al., 2013
Catalase A	*S. cerevisiae*	Multiple targeting signals	Mito, Pex	Petrova et al., 2004
NAD^+^-dependent glycerol 3-phosphat dehydrogenase	*S. cerevisiae*	Protein modification (Phosphorylation)	Pex, Cyt/Nuc	Jung et al., 2010
Glucose-6-phosphat dehydrogenase	*A. thaliana*	Redox dependent heterodimerization	Pex, Plas	Meyer et al., 2011
Alanine-glyoxylate aminotransferase	*H. sapiens*	Protein folding	Cyt, Pex	Fodor et al., 2012
Epoxide hydrolase	*H. sapiens* (liver cells, kidney cells)	Level of expression, quarternary structure	Cyt, Pex	Arand et al., 1991; Enayetallah et al., 2006; Luo et al., 2008

a Abbreviations: Cyt, cytosol; Mito, mitochondrion; Nuc, nucleus; Pex, peroxisome; Plas, plastid.

### Dual targeting of peroxisomal proteins by gene duplication.

Enzymes that are part of shuttle systems to maintain homeostasis between organelles and the surrounding cytoplasm usually display dual targeting. E.g. redox homeostasis is reached by exchange of reduced small intermediates with their oxidized counterparts. In mitochondria, recycling of NAD^+^/NADH is achieved via a malate/aspartate shuttle system (Bakker et al., 2001). In peroxisomes, regeneration of NAD^+^ during β-oxidation also depends on a related malate shuttle since the peroxisomal membrane is permeable for small molecules but impermeable for NADH (van Roermund et al., 1995; Antonenkov et al., 2004).

In *Saccharomyces cerevisiae* three genes encoding malate dehydrogenases have been identified. These code for distinct isoforms, which localize in the cytoplasm, mitochondria and peroxisomes, respectively (McAlister-Henn and Thompson, 1987; Minard and McAlister-Henn, 1991; Steffan and McAlister-Henn, 1992). The different isoenzymes not only carry different targeting signals, but also fulfill distinct functions and show specific regulation. All three malate dehydrogenases are involved in maintaining redox homeostasis. In addition, mitochondrial Mdh1 participates in the tricarboxylic acid cycle, cytosolic Mdh2 is required for gluconeogenesis and peroxisomal Mdh3 is an essential component of the glyoxylate cycle (McAlister-Henn and Thompson, 1987; Minard and McAlister-Henn, 1991; Steffan and McAlister-Henn, 1992)

NADP^+^/NADPH homeostasis in peroxisomes and mitochondria is reached via an alternative shuttle that involves the exchange of isocitrate with alpha-ketoglutarate. This reaction is catalyzed by NADPH dependent isocitrate dehydrogenase (Idp). Similar to malate dehydrogenase, discrete genes (*idp1*, *idp2* and *idp3*) encode the mitochondrial, cytosolic and peroxisomal isoforms of Idp in *S. cerevisiae* (Haselbeck and McAlister-Henn, 1991; Loftus et al., 1994; Henke et al., 1998; van Roermund et al., 1998). Also, the mitochondrial (Cit1) and peroxisomal isoforms (Cit2) of citrate synthase are encoded by different genes in *S. cerevisiae* (Kim et al., 1986; Rosenkrantz et al., 1986; Lewin et al., 1990).

In yeast this type of multiple localization might be preferred since this species has undergone a large scale genome duplication during its evolution (Kellis et al., 2004). This allowed to attribute different cellular functions and localizations to these paralogous genes. In addition encoding isozymes by separate genes facilitates differential control and regulation not only on the transcriptional level, but also at the level of enzyme kinetics and allosteric control. It has been shown that in *S. cerevisiae*, which only contains a very limited number of introns, protein composition is nearly exclusively regulated by differential transcription (Goffeau et al., 1996).

In contrast, other eukaryotes make extensive use of post-transcriptional processes such as alternative splicing to adapt the proteome to environmental changes (Nilsen and Graveley, 2010).

### Dual localization resulting from different transcripts derived from a single gene

In contrast to budding yeast, where gene duplication is commonly used for dual targeting of isozymes, other species often use single genes to produce dually targeted proteins. In *Aspergillus nidulans* synthesis of cytoplasmic, mitochondrial and peroxisomal isoforms of NADP-dependent Idp results from alternative use of transcription initiation sites of the *idpA* gene (Szewczyk et al., 2001). The longer transcript encodes a protein which contains both an N-terminal mitochondrial targeting sequence (MTS) and a C-terminal PTS1. Downstream transcription initiation leads to a shorter *idpA* transcript coding for a protein without the MTS (Szewczyk et al., 2001). While the longer form is preferentially located in mitochondria, the shorter form lacking the MTS is targeted both to peroxisomes and the cytosol. The functional dominance of the N-terminal mitochondrial over the C-terminal peroxisomal targeting signal is most likely due to commitment to mitochondrial import occurring co-translationally before the C-terminus is synthesized (Danpure, 1997).

Differential splicing is another mechanism to generate compartment-specific isoforms from single genes (Yogev and Pines, 2011). In the yeast *Yarrowia lipolytica* the cytoplasmic and peroxisomal isoforms of malate dehydrogenase are generated from alternatively spliced transcripts that differ in their intron size by only four nucleotides. The resulting proteins are highly similar but only one of the Mdh isoenzymes carries a functional PTS1 (Kabran et al., 2012). A related mechanism has been reported for dual targeting of 6-phosphogluconate dehydrogenase (Gnd1) in *Candida albicans*. In this human pathogenic fungus, alternative splicing of *gnd1* transcripts leads to expression of a PTS2 containing isoform (Strijbis et al., 2012). Gnd1 is an essential enzyme of the oxidative branch of the pentose phosphate pathway. This pathway is used to generate NADPH and predominantly resides in the cytosol but has also been found in peroxisomes (Antonenkov, 1989; Corpas et al., 1998; Frederiks and Vreeling-Sindelarova, 2001; Boren et al., 2006; Reumann et al., 2007). Two other enzymes of this pathway, the glucose-6-phosphate dehydrogenase Zwf1 and the 6-phosphogluconolactonase Sol3, have been observed in peroxisomes in *C. albicans* (Strijbis et al., 2012). Differential splicing allows for regulation of dual targeting. In pumpkin leaves the ratio of peroxisomal and cytosolic isoforms of hydroxypyruvate reductase is achieved by light dependent differentially splicing (Hayashi et al., 1996; Mano et al., 1999, 2000).

An unexpected case of dual targeting by alternative splicing was recently described for fungal enzymes involved in glycolysis. This metabolic pathway is considered to be cytoplasmic and glycolytic proteins such as glyceraldehyde-3-phosphate dehydrogenase (GAPDH) and phosphoglycerate kinase (PGK) often serve as cytoplasmic marker proteins in cell biology. A notable exception are trypanosomes, which have transferred the cytoplasmic glycolytic pathway into peroxisome-derived glycosomes (Opperdoes and Borst, 1977). This is considered as an adaptation to the unique lifestyle of these parasites in the bloodstream of vertebrates (Michels et al., 2006). In the basidiomycetous fungus *Ustilago maydis* a C-terminal extended peroxisomal isoform of GAPDH is expressed from an alternatively spliced transcript (Freitag et al., 2012). Inspection of other fungal species revealed that dual targeting of glycolytic enzymes is widespread. In the ascomycetous fungus *Aspergillus nidulans* the peroxisomal isoform of PGK but not of GAPDH is generated by differential splicing (Freitag et al., 2012).

### Dual localization via “leaky” start and stop codons

Further bioinformatic analysis of fungal genes coding for glycolytic enzymes revealed a novel molecular mechanism for dual targeting. Peroxisomal targeting of GAPDH, PGK and triose phosphate isomerase (TPI) is reached by ribosomal read-through of stop codons resulting in a fraction of C-terminally extended proteins ending with a PTS1 (Freitag et al., 2012). Stop codon read-through has been described for retroviral systems where it is used to enlarge protein diversity, but was also observed for cellular transcripts (Bertram et al., 2001; Jungreis et al., 2011). The efficiency of ribosomes to recognize stop codons is affected by sequence context and RNA secondary structure and might be subject of control (Bertram et al., 2001). Isoforms generated by ribosomal read-through correspond to a single transcript and escape detection by transcriptomics. Therefore, this novel mode of dual targeting is difficult to observe.

A related way to produce different isoforms from a single transcript is the use of alternative start codons. The iron-containing SOD of the dinoflagellate *Lingulodinium polyedrum* catalyzes dismutation of superoxide radicals to hydrogen peroxide and oxygen as the first line of defense against reactive oxygen species (ROS) (Bodyl and Mackiewicz, 2007; McCord and Fridovich, 1969). Translation initiation at the first start codon results in an SOD, which contains both an N-terminal targeting signal for plastids and mitochondria and a C-terminal PTS1. This form was suggested to reside in plastids and in mitochondria. Efficient peroxisomal targeting of SOD appears to depend on leaky ribosomal scanning and initiation at a downstream in-frame start codon resulting in an isoform lacking the N-terminal signal sequence (Bodyl and Mackiewicz, 2007). In watermelon cotyledons the mRNA molecules of a 70-kDa heat shock protein contain two in frame start codons. Translational initiation at the first start codon leads to a longer isoform, which carries a N-terminal presequence mediating plastid import, while the shorter isoform localizes to peroxisomes due to a PTS2 (Wimmer et al., 1997).

### Dual targeting of proteins with multiple targeting signals

Although it is commonly assumed that N-terminal signal sequences are dominant over PTS1 (Danpure, 1997), several examples are known where substantial peroxisomal targeting occurs even in the presence of an N-terminal MTS. 3-Hydroxy-3-methylglutaryl coenzyme A lyase (HL) catalyzes the conversion of β-hydroxy-β –methylglutaryl-CoA to acetoacetate, which is important during sterol biosynthesis in mitochondria. In human liver cells HL shows dual localization in mitochondria and peroxisomes. Peroxisomal HL still contains the N-terminal mitochondrial signal sequence, suggesting that dual localization of HL results from an intricate balance between mitochondrial and peroxisomal uptake (Ashmarina et al., 1999).

Another protein with competing signals is type II NAD(P)H dehydrogenase. This enzyme is typically located at the inner mitochondrial membrane but is also found in chloroplasts or peroxisomes (Xu et al., 2013). Three of the seven *Arabidopsis thaliana* genes encoding type II NAD(P)H dehydrogenases (ND) give rise to proteins which are dually targeted both to mitochondria and peroxisomes. These proteins carry an additional C-terminal signal for peroxisomal targeting. Intracellular distribution of the ND proteins with competing signals was shown to depend on the affinity of their signal sequences for their respective receptors/chaperones (Carrie et al., 2008).

If proteins contain competing targeting signals, localization studies with fluorescent proteins may result in ambiguous results. A number of *A. thaliana* acyl-activating enzymes localize either to peroxisomes or to other compartments depending on whether the fluorescent reporter protein was fused at the N- or the C-terminus (Hooks et al., 2012). Therefore it is still unclear whether these proteins occur outside of peroxisomes also in the natural situation.

Competition between the two targeting signals may also be affected by environmental factors. Catalase A (Cta1) of *S. cerevisiae* contains in addition to a non-canonical mitochondrial targeting signal, two peroxisomal targeting signals, an internal signal and a C-terminal PTS1 (Petrova et al., 2004). Both PTSs were shown to be sufficient to target Cta1 to peroxisomes (Kragler et al., 1993). The distribution of Cta1 between peroxisomes and mitochondria is influenced by growth conditions. In the presence of nutrients enhancing H_2_O_2_ formation, like oleic acid, catalase A is predominantly targeted to peroxisomes. In contrast, cultivation of yeast in raffinose leads to increased mitochondrial localization of Cta1. However, the molecular base for this differential targeting is still obscure (Petrova et al., 2004).

### Proteins carrying regulated or inefficient PTS

In all examples discussed above, multiple targeting signals are involved in dual localization residing either concomitantly in a single polypeptide or in different isoforms. In the case of proteins that occur both in peroxisomes and the cytoplasm alternative mechanisms may operate. Dual localization can also result from modified or weak PTS1 signals leading to inefficient import into peroxisomes. Partial peroxisomal localization is difficult to visualize with fluorescent marker proteins, since cytoplasmic fluorescence usually prevents detection of the peroxisomal localization. Therefore this type of dual targeting is likely to be missed in microscopic studies. For the similar case of partial mitochondrial targeting a lacZ-complementation assay has been successfully applied to verify dual targeting (Ben-Menachem et al., 2011). Photobleaching of the cytosolic fraction can also be used to visualize partial peroxisomal localization (Buch et al., 2009).

In the NAD^+^-dependent glycerol 3-phosphate dehydrogenase (Gpd1) of *S. cerevisiae*, protein modification via phosphorylation is used to interfere with peroxisomal targeting (Jung et al., 2010). Gpd1 catalyzes the conversion of dihydroxyacetone phosphate (DHAP) to glycerol 3-phosphate (G3P) to cope with osmotic stress (Merkel et al., 1982; Chen et al., 1987). Gpd1 harbors an N-terminal PTS2, however, the subcellular distribution of Gpd1 depends on environmental factors. Upon osmotic cell stress, Gpd1 is relocated to both the cytosol and the nucleus. This altered localization is triggered by phosphorylation of two serine residues close to the PTS2, thus impairing peroxisomal import (Jung et al., 2010). In *A. thaliana*, peroxisomal import of glucose-6-phosphate dehydrogenase (G6PD1) is triggered by redox signaling and results in relocalization of G6PD1 from chloroplasts to peroxisomes. In this case, formation of a disulfide bridge allows recognition of an internal PTS (Meyer et al., 2011).

A weak/non-canonical PTS1 motif has recently been shown to be critical for proper folding of a PTS1 bearing protein due to prolonged duration of cytosolic localization prior to transfer into peroxisomes (Williams et al., 2012). Especially the import of proteins bearing non-canonical PTS1 motifs may depend on correct protein folding. Even minor misfolding may result in cytosolic localization as was demonstrated for the alanine-glyoxylate aminotransferase (AGT) of humans (Fodor et al., 2012). AGT is known to exhibit a variable distribution in mitochondria and/or peroxisomes in a variety of mammalian species (Danpure, 1997). Similarly, the non-canonical PTS1 motif of human epoxide hydrolase triggers peroxisomal import as a function of concentration and quaternary structure of the protein (Arand et al., 1991; Enayetallah et al., 2006; Luo et al., 2008).

Recent comprehensive studies of the peroxisomal proteome revealed additional proteins that have been previously annotated as cytosolic. Especially in plant peroxisomes, a variety of proteins with unconventional PTS1-motifs has been identified (Reumann et al., 2007; Reumann, 2011). Some of these proteins turned out to reside exclusively in peroxisomes, while others localize in the cytoplasm as determined by microscopy (Reumann et al., 2009). But this does not prove that these proteins are cytosolic since a minor fraction may reside in peroxisomes. At least for one of these proteins, a glutathione reductase, carrying a quite unusual PTS1 (-TNL), partial targeting to peroxisomes was demonstrated (Kataya and Reumann, 2010).

In fungi, proteome studies confirmed the partial peroxisomal localization of glycolytic enzymes and revealed a further candidate, fructose-bisphosphate aldolase (FBA) (Kiel et al., 2009; Managadze et al., 2010). Partial peroxisomal targeting of FBA is presumably mediated by a conserved C-terminal non-canonical PTS1-like motif (Kiel et al., 2009; Freitag et al., 2012). In *U. maydis*, this motif is able to trigger complete peroxisomal import if fused as a dodecamer to GFP, while a full-length GFP-FBA fusion protein results in cytoplasmic fluorescence. This suggests that partial peroxisomal import requires additional features of the protein that interfere with recognition of the unconventional PTS1. The combination of bioinformatic and experimental strategies revealed a heterogeneity of functional PTS1 motifs both in plants and fungi (Reumann, 2011; Freitag et al., 2012). Taken together these data indicate that partial peroxisomal targeting may occur more often than previously assumed. It has even been suggested that all cytosolic proteins may be found in any organelle at least in tiny amounts probably due to mistargeting. This hypothesis was proposed to explain the transfer of whole metabolic pathways from one compartment to another during evolution e.g. that of glycolysis in trypanosomes (Martin, 2010).

## Concluding remarks

The large variety of mechanisms leading to dual targeting of peroxisomal proteins (summarized in Figure 1) suggests that the metabolic capacity of peroxisomes might have been underestimated in the past. This idea is supported by the recent discovery of several hitherto unrecognized peroxisomal metabolic pathways. These include glycolysis in fungal peroxisomes, biotin synthesis in plants and fungi, as well as biosynthesis of secondary metabolites such as siderophores and antibiotics (Bartoszewska et al., 2011; Magliano et al., 2011; Tanabe et al., 2011; Freitag et al., 2012; Grundlinger et al., 2013). Especially for mammals, knowledge of the role of peroxisomes appears to be far from being complete (Schrader and Fahimi, 2008; Islinger et al., 2012). We envision that also in mammals the metabolic capacity of peroxisomes may be of greater variability with dual targeting playing a growing role.

### Conflict of interest statement

The authors declare that the research was conducted in the absence of any commercial or financial relationships that could be construed as a potential conflict of interest.
